# IGF2 role in adrenocortical carcinoma biology

**DOI:** 10.1007/s12020-019-02033-5

**Published:** 2019-08-04

**Authors:** Sofia S. Pereira, Mariana P. Monteiro, Madalena M. Costa, Ângela Moreira, Marco G. Alves, Pedro F. Oliveira, Ivana Jarak, Duarte Pignatelli

**Affiliations:** 1 Instituto de Investigação e Inovação em Saúde (I3S), Universidade do Porto, Porto, Portugal; 2 Institute of Molecular Pathology and Immunology of the University of Porto (IPATIMUP), Porto, Portugal; 3 Endocrine, Cardiovascular & Metabolic Research, Department of Anatomy, Multidisciplinary Unit for Biomedical Research (UMIB), ICBAS, University of Porto, Porto, Portugal; 4 Biology and Genetics of Reproduction, Department of Microscopy, Laboratory of Cell Biology, Multidisciplinary Unit for Biomedical Research (UMIB), ICBAS, University of Porto, Porto, Portugal; 5 Health Sciences Research Center, University of Beira Interior, Covilhã, Portugal; 6 Department of Endocrinology, Hospital S.João, Porto, Portugal

**Keywords:** Adrenocortical tumors, Adrenocortical carcinomas, IGF2, Hallmarks of cancer

## Abstract

**Purpose:**

Clinical outcomes of adrenocortical carcinomas (ACC) could be improved by using novel treatment targets based on the recent advances of tumor biology knowledge. Insulin-like growth factor 2 (IGF2) protein expression is usually 8–80 fold higher in ACC when compared to normal adrenal glands (N-AG) or adrenocortical adenomas (ACA), despite the fact that the biological features of high vs. low IGF2 expressing ACC have not yet been well characterized. Our goal was to understand the IGF2 role in ACC biology by focusing in several cancer hallmarks, including cell proliferation, viability, invasion, and metabolism.

**Methods:**

IGF2 immunohistochemistry expression was evaluated in ACC (*n* = 13), non-functioning adrenocortical adenoma (ACAn) (*n* = 14), and N-AG (*n* = 9). The effects of IGF2 (50, 100 ng/mL) in cell proliferation, viability, invasion, and metabolism, as well as in MAPK/ERK and mTOR pathways activation and N-cadherin expression, were evaluated in the ACC human cell line H295R.

**Results:**

IGF2 expression was increased in ACC compared to ACAn and N-AG. Exposure to 100 ng/mL of IGF2 increased H295R cell proliferation and viability. mTOR inhibition reverted IGF2 triggered cell proliferation and viability while MEK/MAPK/ERK inhibition only reverted IGF2 effects on cell proliferation. IGF2 at a 50 ng/mL concentration increased the glycolytic flux and decreased glutamine consumption.

**Conclusions:**

IGF2 is an excellent marker to differentiate ACC from ACAn. In addition, IGF2 was demonstrated to influence adrenocortical cancer cell proliferation, metabolism, and viability, but not the cell invasion. These data support that different IGF2 concentrations in ACC can be responsible for different biological behaviors of ACC.

## Introduction

Adrenocortical tumors (ACT) are common adrenal cortex tumors that affect 3–10% of the adult population [1]. The majority of ACT are benign, non-functioning and incidentally discovered during imaging studies performed for unrelated clinical reasons [2]. In contrast to adrenocortical adenomas (ACA), adrenocortical carcinomas (ACC) are rare, the majority are diagnosed in an advanced stage and usually have a poor prognosis [1]. Detailed knowledge of molecular alterations that underlie the malignant transformation of benign cells, as well as the identification of the specific cell cycle alterations found in cancer cells that are associated with enhanced survival, have not only allow the identification of new diagnostic and prognostic tools but also provides the potential to disclose novel treatment targets. The IGF2 system is one of the key molecular mechanisms that was recurrently described to be involved in ACC pathophysiology [3–7].

IGF2 is a growth factor secreted mainly by the liver but also in smaller amounts in the majority of tissues where it acts in an autocrine or paracrine way. IGF2 actions are mediated by the IGF1 receptor (IGF1R), insulin receptor (IR) and IGF2 receptor (IGF2R) [8, 9]. IGF2 activates tyrosine kinase receptors that in turn lead to mitogen-activated protein kinase (MAPK) and phosphatidylinositol 3-kinase (PI3K)/Akt pathways activation. Activated Akt is then able to trigger the subsequent activation of the mammalian target of rapamycin (mTOR) pathway. MAPK, PI3K/Akt/mTOR pathways are well-described mechanisms involved in proliferation, survival, and metastasis of cancer cells [8–10].

IGF2 overexpression was previously described as responsible for increased proliferation of ACC cells [11]. However, despite being increased in the majority of the tumors, IGF2 expression can be highly variable in ACC cells. IGF2 mRNA expression was found to be 10–20 fold higher in ACC compared to normal adrenal glands or ACA, while IGF2 protein expression was described to be 8–80 fold greater in ACC than in normal adrenal glands or ACA [11–16]. Therefore, our goal was to extend the understanding of the IGF2 role in ACC evaluating the expression of IGF2 in the adrenocortical tumors and the paracrine effects of IGF2 in the proliferation, viability, invasion, and metabolism of ACC cells.

## Materials and methods

### Adrenal tissue

Adrenal tumor samples were obtained from patients with ACT (*n* = 27), comprising 13 ACC and 14 non-functioning adenomas (ACAn). Normal adrenal glands (N-AG) (*n* = 9) retrieved during nephrectomy performed for urologic conditions from patients without adrenal pathology were used as controls. A summary of the patient’s clinical features and tumors characteristics is present in Table 1. The study was approved by the Ethics Committee of the Centro Hospitalar São João - Porto, Portugal. The participants provided their written informed consent to tumor storage in the tumor bank of the Department of Pathologic Anatomy—Centro Hospitalar São João, Porto, for later research use.

**Table 1:** Patients clinical features and tumors characteristics

	ACC (*n* = 13)	ACAn (*n* = 14)
Median age in years (Range)	46 (27–59)	49 (23–76)
Tumor size (mm)	188 ± 98	38 ± 23
Weiss score	>4	≤2
Tumors functionality (Functioning/non-functioning)	8/5	0/14
ENSAT stage (I, II, III, IV)	(0/1/8/4)	NA
p53 (% stained area)	5.3 ± 1.91	3.5 ± 0.45
β-catenin localization (M:C:N)^#^	3:8:2	0:8:6

*ACC* adrenocortical carcinomas, *ACAn* non-functioning adrenocortical adenoma, *NA* field is not applicable, *ENSAT* European Network for the Study of Adrenal Tumours, *# M* only membrane, *C* Membrane + cytoplasm, *N* Membrane + cytoplasm + nucleus

### Immunohistochemistry (IHC) and data analysis

IHC was performed in 3 μm formalin-fixed paraffin-embedded tissue sections mounted on adhesive microscope slides. Sections were deparaffinized, rehydrated in graded alcohols and underwent antigen retrieval performed by microwave treatment in 0.01 M-citrate buffer at pH 6.0, during 9 min. The sections were then incubated overnight at 4 °C with the primary antibody against IGF2 (Table 2). The detection of the immune reaction was performed using the avidin-biotin-peroxidase method (1:100; Vector Laboratories, Inc., Peterborough, UK). DAB (3,3′- diaminobenzidine) was used as chromogen and hematoxylin as nuclear counterstaining. Placental tissue was used as positive control, while omission of the primary antibody from incubation was used as negative control.

**Table 2:** Antibodies used in this study

Antibodies	Source	Reference and vendor	Dilution
*Primary antibody used in Immunohistochemistry*			
IGF2	Rabbit	Ref. ab9574; Abcam, Cambridge, United Kingdom	1:100
β-catenin	Rabbit	Ref. 424A-14; Cell Marque, Rocklin, CA, USA	1:500
p53	Rabbit	Ref. 453M-94; Cell Marque, Rocklin, CA, USA	1:100
*Primary antibody used in Immunofluorescence*			
BrdU	Mouse	Ref. sc-32323; Santa Cruz Biotechnology, Heidelberg, Germany	1:200
N-cadherin	Rabbit	Ref. ab18203; Abcam, Cambridge, United Kingdom	1:200
p21	Mouse	Ref. SC-6246; Santa Cruz Biotechnology, Heidelberg, Germany	1:100
*Primary antibodies used in western blot*			
Phospho-ERK 1/2	Rabbit	Ref. 4370S; Cell Signaling Technology, Danvers, USA	1:2000
Total-ERK 1/2	Mouse	Ref. 4696S; Cell Signaling Technology, Danvers, USA	1:2000
N-cadherin	Rabbit	Ref. ab18203; Abcam, Cambridge, United Kingdom	1:1000
β-actin	Goat	Ref. sc1616; Santa Cruz Biotechnology, Heidelberg, Germany	1:250
*Secondary antibody used in immunohistochemistry*			
Biotinylated anti-rabbit	Swine	Ref. EO35301-2; Dako, Glostrup, Denmark	1:200
*Secondary antibody used in immunofluorescence*			
Anti-mouse IgG (H + L), Alexa Fluor® 488	Goat	Ref. 4408; Cell Signaling Technology, Danvers, USA	1:1000
Anti-rabbit IgG (H + L), Alexa Fluor® 555	Goat	Ref. 4413; Cell Signaling Technology, Danvers, USA	1:1000
*Secondary antibodies used in western blot*			
Anti-goat IgG-HRP	Donkey	Ref. sc-2020; Santa Cruz Biotechnology, Heidelberg, Germany	1:1000
Anti-mouse IgG-HRP	Goat	Ref. 12–349; Merck-Millipore, California, USA	1:2000
Anti-rabbit IgG-HRP	Goat	Ref. ab6721; Abcam, Cambridge, United Kingdom	1:2500

*IGF2* insulin-like growth factor 2, *BrdU* 5-bromo-2-deoxyuridine, *ERK* extracellular signal-regulated kinase, *HRP* horseradish peroxidase, *Ig* immunoglobulin

Immunohistochemistry for p53 and β-catenin staining was performed as previously reported [17].

From each section slide, a minimum of 10 microphotographs were taken (Leica EC3 camera, Leica, Germany) and images were analyzed using the software ImageJ (originated at the National Institutes of Health, USA) that allows separation of the stained area from the total area in order to calculate the percentage of the area stained both for IGF2 and p53. The staining for β-catenin exhibited different cell distributions (cell membrane, cytoplasm, and nucleus), and so the distribution of β-catenin staining was evaluated by direct observation.

### Cell culture

Human adrenocortical carcinoma cell line (H295R) obtained from CLS Cell Lines Service GmbH (Eppelheim, Germany) was cultured in Dulbecco’s Modified Eagle Medium: Nutrient Mixture F-12 (DMEM/F12; Sigma-Aldrich, St Louis, MO, USA) supplemented with 0.365 g/L of l-glutamine (Sigma-Aldrich, St Louis, MO, USA), 10 mL/L of penicillin-streptomycin (Sigma-Aldrich, St Louis, MO, USA), 2.5% of NuSerum (BD Bioscience, San Jose, CA) and 1% of Insulin-Transferrin-Selenium Premix (ITS) (Corning, NY, USA). The medium was changed three/four times per week and the cells were detached for sub-culturing with a 0.25% trypsin-ethylenediaminetetraacetic acid (EDTA) solution (Sigma-Aldrich, St Louis, MO, USA). Cell cultures were handled in a laminar flow chamber and maintained at 37 °C in an incubator (Heracell 150i, Thermo Scientific, Waltham, MA USA) with 5% CO_2_. Before incubating cells with any of the used growth factors or inhibitors, a serum and ITS 2 h starvation was performed to reduce the basal signaling activity and washout the insulin from the media since insulin can signal through the IGF1-R [18] and so interfere with our results. The starvation time was optimized based on the need to balance the evaluation of the effects on signaling pathways analyzed while ensuring cell viability. All the experiments were also performed without serum or ITS, by the same reasons appointed before.

Cells were then incubated with 2 different IGF2 concentrations (50 ng/mL and 100 ng/mL) for 24 h, except when the aim was to evaluate the activation of the MAPK/extracellular signal-regulated kinases (ERK) pathway or the mTOR pathway. In those cases, the incubations were performed for 5, 10, and 20 min.

In addition, as H295R cells in cell culture are capable of secreting IGF2 in amounts increasing along time [19], we decided not to exceed a total of 48 h of total culture time (24 h without treatment +24 h with treatment) in order to mitigate the putative effect of autocrine IGF2 and maintain high cell viability rates in the control group (cells without IGF2).

IGF2 concentrations chosen were based on the previous knowledge that H295R cells produce IGF2 and also on the fact that IGF2 levels in ACC can vary more than ten times [12, 19, 20]. So, the in vitro study was designed in order to attain a spectrum of IGF2 in the medium ranging from 5 (50 ng/mL) to 10 times (100 ng/mL), approximately. Moreover, the volume on the wells was adapted to ensure that the desired IGF2 concentration was achieved.

The MEK inhibitor (PD184352, Sigma-Aldrich) concentration used (10 µM) was previously reported to significantly decrease the phospho-ERK in H295R cells [21]. For mTOR inhibitor (Rapamycin, Alfa Aesar, MA, USA), two concentrations (50 and 100 nM) were evaluated, using a mTOR Elisa Kit described below. The higher concentration showed to be more effective on the mTOR expression inhibition. Besides, this concentration was successfully used before in this cell type [22].

For cell proliferation, viability and metabolism, each treatment was replicated five times. The cell invasion, senescence, and N-cadherin analysis were replicated three times.

### Cell proliferation assay

H295R cells (0.4 × 10^6^ cells/well) were cultured in 24-well-plates with complete medium for 22 h followed by a 2 h period with serum and ITS depleted medium. H295R cells were then incubated for 24 h in the presence of IGF2 alone, IGF2 + MEK inhibitor (PD184352), IGF2 + mTOR inhibitor (Rapamycin) or IGF2 + MEK inhibitor + mTOR inhibitor. H295R cell proliferation was monitored by 5-bromo-2-deoxyuridine (BrdU, 10 µM, Sigma-Aldrich) incorporation over a 2 h period. Cultured cells were harvested by cyto-spinning, fixed in 4% paraformaldehyde (Merck Millipore, Darmstadt, Germany) followed by immunofluorescence for BrdU staining (Table 2). A minimum of 500 cells were counted at a 400x magnification.

### Cell viability assay

H295R cells (0.05 × 10^6^ cells/well) were cultured in 96 well-plates with complete medium for 22 h followed by a 2-h period with serum and ITS depleted medium. H295R cells were then incubated for 24 h with IGF2 alone, IGF2 + MEK inhibitor (PD184352), IGF2 + mTOR inhibitor (Rapamycin) or IGF2 + MEK inhibitor + mTOR inhibitor, in the presence of 10% Alamar Blue (Bio-Rad AbD Serotec, Oxford, UK). Absorbance was measured at wavelengths of 570 nm and 595 nm, at 0, 12 and 24 h. The % of resazurin reduction was calculated using the following equation:

$$\% {\it{Reduction}} = \left( {\frac{{Eox_{\lambda 2}xA_{\lambda 1} - Eox_{\lambda 1}xA_{\lambda 2}}}{{Ered_{\lambda 1}xA_{blank\lambda 2} - Ered_{\lambda 2}xA_{blank\lambda 1}}}} \right)x100,$$

where λ1 = 570 nm, λ2 = 595 nm, Eox_1_ = 80,573, Eox_2_ = 117,216, Ered_1_ = 155,667 and Ered_2_ = 14,652.

### p21 immunofluorescence

H295R cells (0.8 × 10^6^ cells) were incubated in coverslips immersed in 6 well-plates with complete medium for 22 h followed by 2-hour period with serum and ITS depleted medium. H295R cells were then exposed to IGF2 with and without the pathway’s inhibitors (Rapamycin for mTOR pathway inhibition and PD184352 for MAPK pathway inhibition). After 24 h, the cells were fixed in 4% paraformaldehyde for 15 min before immunofluorescence was performed. The coverslips were incubated overnight at 4 °C with the primary antibody for p21 (Table 2), followed by the secondary antibody incubation for 45 min to allow p21 detection (Table 2). The slides were then counterstained with 1 µg/ml DAPI (Sigma-Aldrich). Coverslips were mounted in slides with mounting solution (90% glycerol, 0.5% N‑propyl-gallate, 20 nM Tris, pH 8). A minimum of 500 cells were counted at a ×200 magnification.

### Cell nucleus size

After H295R cells incubation with IGF2, with and without the pathways inhibitors (Rapamycin for mTOR pathway inhibition and PD184352 for MAPK pathway inhibition), cells were stained with 1 µg/ml DAPI (Sigma-Aldrich) and the nucleus area was evaluated in a minimum of 100 cells for experimental condition, using the ImageJ software.

### Invasion assay

To evaluate the invasion capacity of H295R cells incubated with different IGF2 concentrations, cell culture inserts with an 8.0 μm pore size membrane (BD Biocoat 24-well Matrigel Chambers, BD Bioscience Bedford, MA, USA) were used according to the manufacturer’s protocol. Matrigel-coated inserts were pre-incubated for 1 h with serum-free DMEM-F12, before H295R cells (0.1 × 10^6^ cells) were seeded in the upper chamber of the well and cultivated in the presence of IGF2. Medium supplemented with 30% Nu-serum was used as chemo-attractant and so added into the bottom of the lower chamber in the 24-well plate. After 24 h, the medium was collected, the membranes of the inserts were fixed with 70% ethanol and the cells were stained with 0.2% crystal violet for 10 min, as previously described [23]. The membranes were then mounted on slides using entellan and the cells that invaded the membrane were observed and counted using an optical microscope (Zeiss AxioPlan microscope, Zeiss, Germany).

### N-cadherin immunofluorescence

The H295R cell (0.4 × 10^6^ cells) were incubated in coverslips immersed in 24-well-plates, with complete medium for 22 h followed by 2-h period with serum and ITS depleted medium (NuSerum). H295R cells were then exposed to IGF2 for 24 h, after which cells were fixed in 4% paraformaldehyde for 15 min before immunofluorescence was performed. The coverslips were incubated overnight at 4 °C with the primary antibody for N-cadherin (Table 2), followed by the secondary antibody incubation for 2 h to allow N-cadherin detection (Table 2). The slides were then mounted and counterstained with Vectashield hardset with Dapi (ref. H1500, Vector Laboratories, UK).

### Western blot

After IGF2 incubation, cell proteins were extracted using RIPA buffer (ref: 20–188, Sigma-Aldrich, USA) with protease inhibitor (ref: 4693124001, Roche, Switzerland) and phosphatase inhibitor (ref: 4906845001, Roche). Extracted proteins were quantified using the Pierce™ BCA Protein Assay Kit (ref: 23225, ThermoFisher Scientific, USA). A total of 20 µg of protein was heated at 95 °C for 10 min, fractionated on a 12% sodium dodecyl sulfate-polyacrylamide gel electrophoresis (SDS-PAGE) and transferred to polyvinylidene difluoride membranes. The membranes were blocked in a Tris-buffered saline solution with 0.05% Tween 20 containing 5% BSA (ref: A7906, Sigma- Aldrich) and incubated overnight at 4 °C with the primary antibodies, separately (Table 2). Mouse β-actin was used as protein loading control for N-cadherin quantification. Immune-reactive proteins were detected separately using the respective secondary antibody (Table 2). Membranes were reacted with ECL detection (ref. 32209, GE Healthcare) system and read with the ChemiDoc™ XRS+ System (Bio-Rad, UK). The densities of each band were obtained using the Quantity One Software (Bio-Rad, UK).

### mTOR Elisa

After H295R cells incubation with different IGF2 concentrations, proteins were extracted and phospho-mTOR and total-mTOR expression were assessed using the RayBio® Human and Mouse Phospho-mTOR (Ser2448) and Total mTOR ELISA Kit (PEL-mTOR-S2448-T-1, RayBio, GA, USA), following the manufacturer’s instructions.

### Nuclear magnetic resonance (NMR) spectroscopy

^1^H NMR spectroscopy (VNMRS 600 MHz, Varian, Inc. Palo Alto, CA) was used to determine metabolite concentrations in H295R cell culture media after IGF2 incubation. Sodium fumarate was used as internal reference (6.50 ppm) to quantify the following metabolites (multiplet, ppm): lactate (doublet, 1.33); alanine (doublet, 1.45); pyruvate (singlet, 2.36); glutamine (triplet, 3.75) and H1-α glucose (doublet, 5.22) as previously described [24]. The relative areas of ^1^H NMR resonances were quantified using the curve-fitting routine supplied with the NUTSpro™ NMR spectral analysis program (Acorn, Fremont, CA, USA) and the results were normalized to the number of cells present at the time when the media was collected.

### Statistical analysis

All results are presented as mean ± standard error (SE). D’Agostinho & Pearson test was used to evaluate variables normality. For continuous variables that passed this test, one-way ANOVA test with the post-hoc Tukey test was used to compare the means of three groups. For the variables that did not pass the normality test, the Kruskal–Wallis with a Post-hoc Dunn’s test was used. The correlations between continuous variables were evaluated using the Pearson Test. The diagnostic accuracy of IGF2 was evaluated using the receiver operating characteristic (ROC) curve. Statistical analysis was performed using the GraphPad Prism version 6.01 for Windows (GraphPad Software, La Jolla, CA, USA). The significance level was defined by a value of *p* < 0.05.

## Results

### IGF2 in tumor and normal human adrenocortical tissues

#### The expression of IGF2 is significantly higher in ACC

**Fig. 1:**
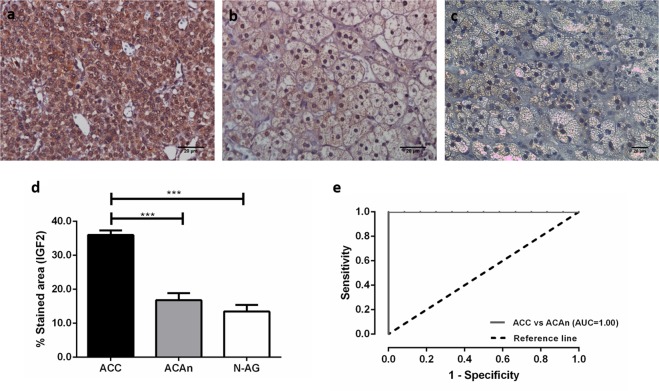
Immunohistochemistry staining for IGF2 (Scale = 20 µm) in adrenocortical carcinoma (ACC) **a**, non-functioning adrenocortical adenoma (ACAn) **b**, and normal adrenal gland (N-AG) **c**. Graphic representation of the percentage of area staining for IGF2 in the studied groups **d** and ROC curves with the respective area under the curve (AUC) to compare carcinomas with adenomas **e** (ANOVA: ***p < 0.001)

The percentage of stained area for IGF2 was significantly higher in ACC (35.97 ± 1.38) compared to ACAn (16.79 ± 2.09) and N-AG (13.45 ± 1.94), *p* < 0.001 (Fig. 1a–d). ROC Curve analysis showed an area under the curve (AUC) of 1.00 for discrimination between ACC and ACAn, with a specificity and sensibility of 100%, using a cut-off of 27.11% of IGF2 stained area (Fig. 1e). No correlation was observed between IGF2 and p53 expression (*R* = 0.47). Furthermore, IGF2 expression was not correlated with the β-catenin localization, in ACC (Table 1).

### In vitro analysis of the influence of IGF2 in the H295R proliferation, viability, invasion, and metabolism

#### A high IGF2 concentration increases H295R proliferation and viability

**Fig. 2:**
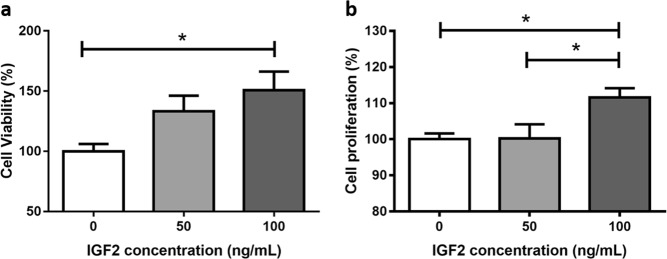
H295R cells viability **a** and proliferation **b** after incubation without or with IGF2 at the concentrations of 50 and 100 ng/mL for 24 h (ANOVA: *p < 0.05)

H295R incubation with the highest IGF2 concentration tested (100 ng/mL) led to a significant increase in cell proliferation (111.6 ± 2.56 %) after 24 h when compared to the cells incubated with the 50 ng/mL concentration (100.2 ± 3.93 %) and to the cells that were not supplemented with IGF2 (100.0 ± 1.61 %; *p* < 0.05). IGF2 (100 ng/mL) also significantly increased cell viability (150.8 ± 15.40 %) when compared to the control (100.0 ± 6.11 %; *p* < 0.05) (Fig. 2).

#### IGF2 increases both phospho-ERK and phospho-mTOR expression

**Fig. 3:**
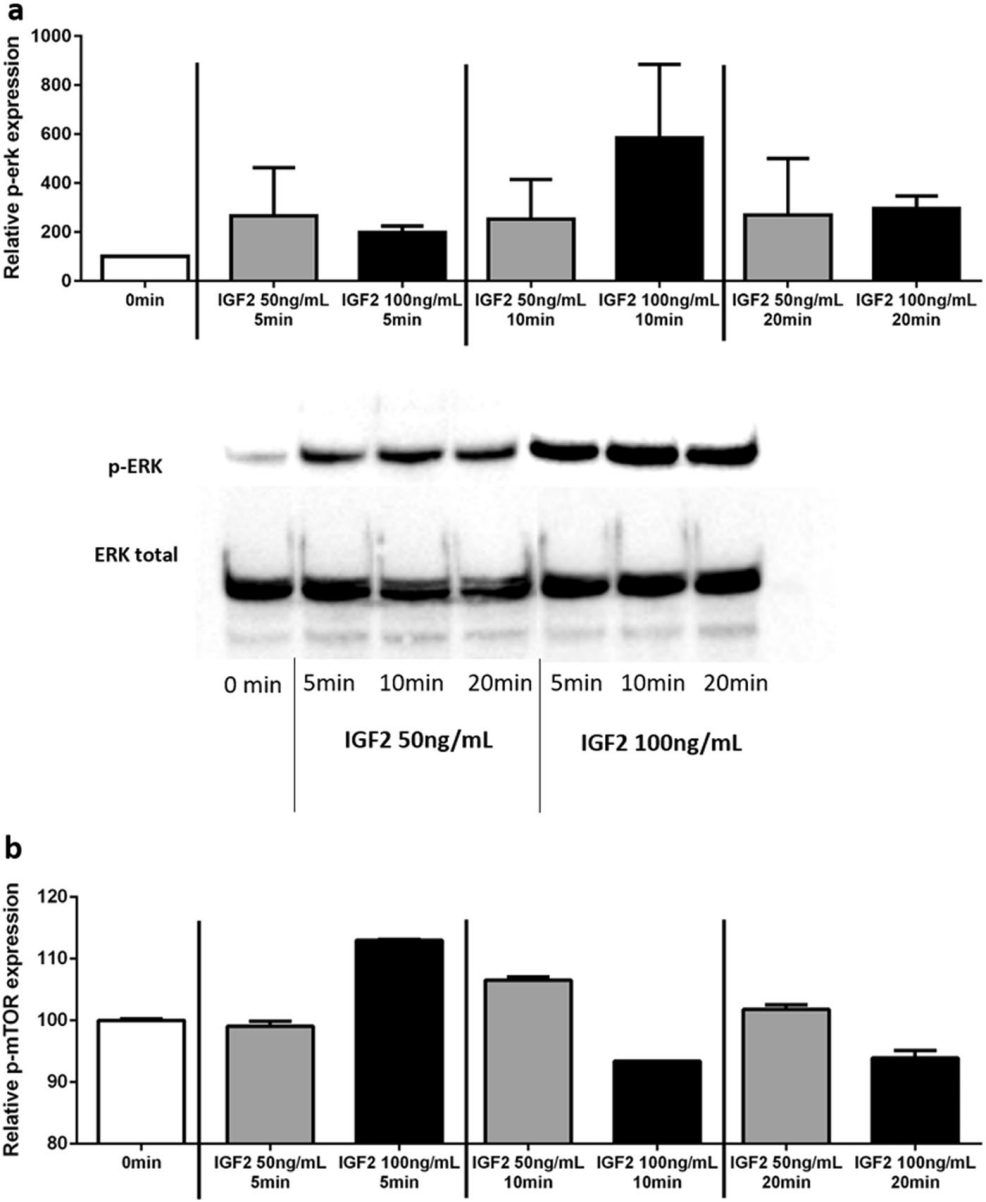
Relative phospho-ERK expression **a** and phospho-mTOR expression **b**, after IGF2 incubation at the concentrations of 50 and 100 ng/mL for 5, 10, and 20 min

Incubation with both concentrations of IGF2 led to a rapid increase of the phospho-ERK expression (5 min: 50 ng/mL: 266.6 ± 138.8: 100 ng/mL: 198.2 ± 18.78) (Fig. 3a). Phospho-ERK expression was stabilized thereafter with the IGF2 concentration of 50 ng/mL, during the study time (5, 10, and 20 min), while the concentration of 100 ng/mL led to higher levels of phospho-ERK expression after 10 min of incubation (584.7 ± 212.3) followed by a subsequent decrease after this time point (20 min:296.2 ± 35.76) (Fig. 3a).

Incubation with 100 ng/mL of IGF2 led to an increase of the phospho-mTOR expression, at 5 min (0 min:100.0 ± 0.18; 5 min: 113.0 ± 0.07), followed by a subsequent decrease after this time point (Fig. 3b). IGF2 at 50 ng/mL only slightly increased the phospho-mTOR expression after 10 min of incubation (113.0 ± 0.07, Fig. 3b).

#### mTOR inhibition reverts IGF2 triggered viability

**Fig. 4:**
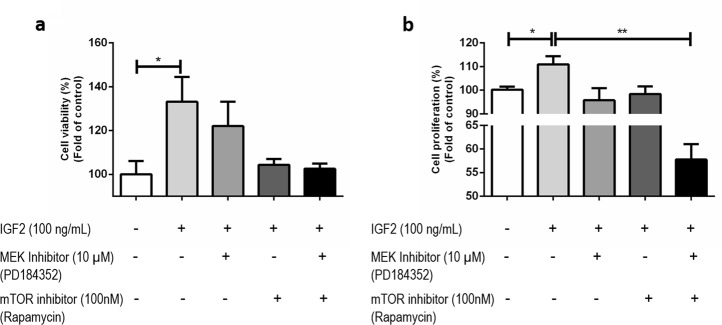
Cell viability **a** and proliferation **b** after treatment with IGF2 (100 ng/mL) with and without the MAPK and mTOR inhibitors (100 nM of Rapamycin for mTOR pathway inhibition and 10 nM of PD184352 for MAPK pathway inhibition) (ANOVA: *p < 0.05)

Co-incubation of IGF2 with the mTOR inhibitor blocked the IGF2-stimulated viability (IGF2 100 ng/mL: 133.1 ± 11.40; IGF2 100 ng/mL + Rapamycin 100 nM: 104.4 ± 2.62) (Fig. 4a) while MEK inhibitor did not influence it (Fig. 4a).

#### MEK and mTOR inhibition reverts IGF2 triggered proliferation

Co-incubation of IGF2 with a MEK inhibitor blocked the IGF2-stimulated proliferation (IGF2 100 ng/mL: 110.9 ± 3.48; IGF2 100 ng/mL + PD184532 10 µM: 95.78 ± 5.10) (Fig. 4b). Similar results were obtained using the mTOR inhibitor (IGF2 100 ng/mL: 110.9 ± 3.48; IGF2 100 ng/mL + Rapamycin 100 nM: 98.41 ± 3.23) (Fig. 4b). MEK and mTOR inhibitor have a cumulative effect on the cell proliferation leading to a reduction of the proliferation of almost 50% when compared with the control group (control: 100.2 ± 1.31; IGF2 100 ng/mL + Rapamycin 100 nM + PD184532 10 µM: 7.76 ± 3.28).

#### mTOR and MEK inhibitors did not influenced H295R cells senescence

p21-positivivity, a cell senescence marker [25] was present in a low number of cells (<5%) independently of the study group. Besides that, the number of p21 positive cells did not vary with the presence of mTOR and/or MEK inhibitors (Supplementary file 1). The nucleus size was also similar in all the experimental groups (Supplementary file 1).

#### IGF2 does not influence cell invasion capacity

**Fig. 5:**
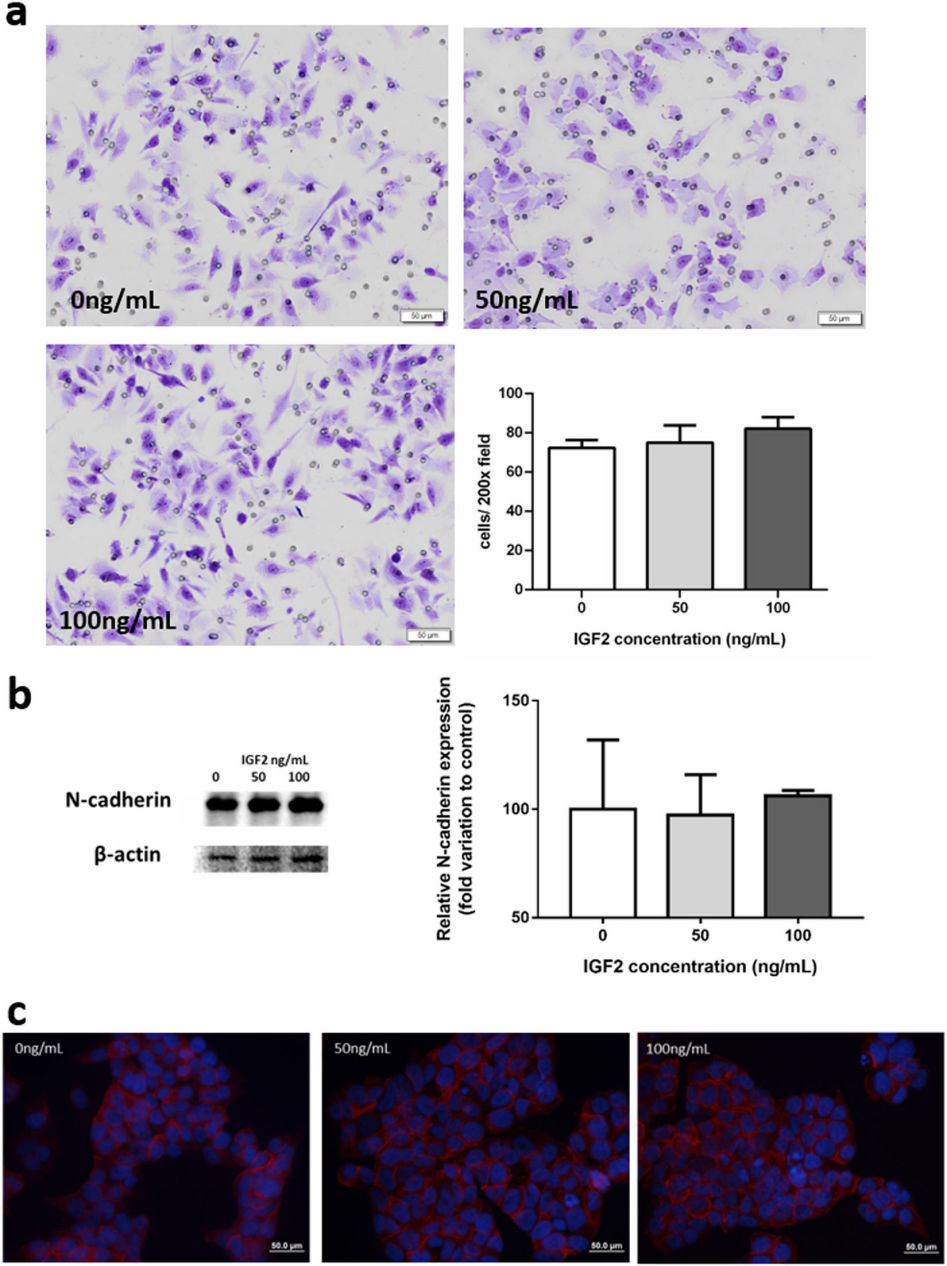
Matrigel membrane invaded with H295R cells **a**. N-cadherin expression after 24 h incubation with IGF2 at the 50 and 100 ng/mL concentrations evaluated by western blot **b** and immunofluorescence **c**

H295R cells invasion capacity was not influenced by IGF2 incubation. Besides that, IGF2 did not influence H295R cells N-cadherin expression (Fig. 5).

#### IGF2 incubation at a concentration of 50 ng/mL decreased glutamine consumption

**Fig. 6:**
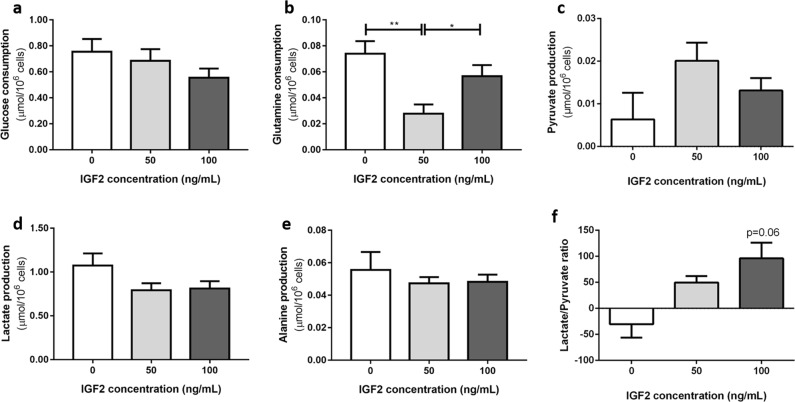
Glucose **a** and glutamine **b** consumption; pyruvate **c**, lactate **d** and alanine **e** production; lactate/pyruvate ratio **f** after IGF2 incubation at the concentrations of 50 and 100 ng/mL (ANOVA: *p < 0.05; **p < 0.01; *p* = 0.06: 0 ng/mL vs 100 ng/mL)

Glutamine consumption by H295R cells was significantly lower after IGF2 incubation at a concentration of 50 ng/mL (0.03 ± 0.01 µmol/10^6^ cells) compared to control (0.07 ± 0.01 µmol/10^6^ cells, *p* < 0.01) or to cells incubated with the IGF2 concentration of 100 ng/mL (0.06 ± 0.01 µmol/10^6^ cells, *p* < 0.05, Fig. 6). Besides that, IGF2 dose-dependently increased the lactate/pyruvate ratio (0 ng/mL: −30,43 ± 25.70; 50 ng/mL: 49.68 ± 12.25; 100 ng/mL: 96.14 ± 30.27, Fig.6f).

Glucose consumption and pyruvate, lactate and alanine production were not significantly influenced by IGF2 incubation (Fig. 6).

## Discussion

The IGF2 system represents a crucial pathway in the tumor biology of ACC and was considered an attractive target for the treatment of these tumors [8]. IGF2 protein expression was found to be 8–80 fold higher in ACC when compared to the N-AG or ACA, although the biological differences between high and low IGF2 expressing ACC have not yet been well characterized [11–16]. In order to gain further insight into the role of IGF2 in ACC pathogenesis, after having analyzed the IGF2 expression in different ACT and N-AG, we have evaluated the effects of IGF2 in some important hallmarks of cancer, including cell proliferation, viability, invasion, and metabolism in vitro in a human adrenocortical cancer cell line.

As previously reported, IGF2 expression was found to be significantly higher in ACC compared with ACA [11, 14, 15, 17, 26]. Besides that, our study showed that IGF2 presents a 100% of specificity and sensitivity to distinguish ACC from ACAn. So, using a computerized morphometric analysis of IGF2 expression may be used as a biomarker for the differential diagnosis between ACA and ACC. Thus, the potential use of IGF2 expression as a valuable diagnostic marker is herein demonstrated, whether this molecular marker could also provide additional information on the tumor biological behavior and disease progression, so that this could be used as a prognostic marker as well, is currently unknown. Nevertheless, in a previous work by other authors, the percentage of IGF2 stained cells was not shown to influence ACC survival [11].

Since β-catenin and p53 have been demonstrated to have an important role in ACC biology, we decided to assess whether there was a correlation between IGF2 and those proteins’ expression. Although no mutational analysis was performed in this study, a small percentage of ACC exhibited a very high p53 expression due to accumulation of this protein in the cell, which is suggestive of the presence of p53 mutations [27]. Besides that, abnormal β-catenin localization i.e., in the cytoplasm and/or the cell nucleus instead of the normal localization at the cell membrane was also evaluated [28]. Our study showed that IGF2 expression in ACC is not correlated either with β -catenin or with p53 status.

The high variability in IGF2 protein expression in ACC observed by several authors, raises the hypothesis that this could be reflected in distinct biological behaviors. To test this hypothesis a human adrenocortical carcinoma cell line (H295R) was used to assess the influence of IGF2 in several cancer hallmarks.

IGF2 at the higher concentration tested increased H295R cell proliferation and viability. Since the same IGF2 concentration also increased phospho-ERK and phospho-mTOR expression, we decided to investigate whether MAPK/ERK or mTOR pathways could be responsible for the increased cell proliferation and viability. The use of the MEK inhibitor was only able to revert IGF2 triggered cell proliferation, while cell viability was virtually unaltered. On the other side, mTOR inhibition was able to revert both IGF2 triggered cell proliferation and viability. These data supports that although both pathways are triggered by IGF2 binding to IGF1R [8], MAPK/ERK pathway is involved in the proliferative increment but does not seem to be involved in mediating the IGF2 effects in cell viability, while PI3K/Akt/mTOR pathway seems to be involved in the regulation of both processes.

Similarly, previous preclinical studies using different mTOR inhibitors also showed a decrease in ACC cell proliferation and ACC xenographs growth [29–32]. Unfortunately, despite these promising preclinical findings, patients with advanced ACC treated with a mTOR inhibitor (everolimus) showed no clinically significant benefits [33]. Some studies proposed that other IGF1R signaling pathways, such as MAPK/ERK pathway could be activated during the treatment in order to overcome the mTOR inhibitors effects [30, 33]. In our study, we showed that the combination of both inhibitors had a powerful effect reducing H295R cells proliferation and so, it supports the hypothesis that, MAPK/ERK and mTOR inhibitors combination could be a possible treatment for ACC that should be tested in the future.

Neither matrigel invasion nor N-cadherin expression were affected in H295R cells treated with IGF2. These results point out that IGF2 does not seem to affect cell invasiveness and in fact are consistent with reports that the overall and the disease-free survival in ACC patients is unrelated to IGF2 expression [11].

Finally, our data show that IGF2 is able to interfere in ACC cell metabolism by dose-dependently increasing the lactate/piruvate ratio that is a robust indicator of the anaerobic metabolism and the redox status of the tissue. Besides that, different IGF2 concentrations influenced differentially the glutamine consumption. Glutamine is known to enter the cell through the Alanine–Serine–Cysteine transporter (ASCT2). When inside the cell, glutamine by itself can contribute to nucleotide biosynthesis or it can suffer glutaminolysis, a metabolic pathway in which glutamine is catabolized to generate ATP and lactate. For that glutamine is initially converted by glutaminase, into glutamate which is then converted to α‑ketoglutarate, a tricarboxylic acid (TCA) cycle intermediate, to produce both ATP and anabolic carbons for the de novo synthesis of amino acids, nucleotides, and lipids [34, 35]. IGF2 at a lower concentration (50 ng/mL) seems to lead to a compensatory balance between the anaerobic metabolism and glutamine consumption, as the anaerobic metabolism is increased while glutaminolysis decreases. IGF2 at a higher concentration (100 ng/mL) leads to similar levels of anaerobic metabolism with increased levels of glutamine consumption when compared to the lower concentration (50 ng/mL), suggesting that at a higher concentration IGF2 leads to cumulative effects on both pathways instead of compensatory.

The major limitation of this study is the small number of tumor samples that could be used and the fact that all the functional studies are based on a single cell model. This limitation was rather inevitable as ACC are relatively rare and H295R is the only well-characterized cell line that can be used as a model for this malignancy. In order to overcome this limitation, it is our plan to perform ACC primary cell cultures to validate these results in the future. Besides that, H295R cells have a specific genetic profile that includes the presence of beta-catenin protein accumulation associated with a point mutation of Ser45 in exon 3 and so it must be stressed that these results may only be applied to tumors with this characteristic.

In conclusion, our results demonstrate that morphometric computerized analysis of may possibly be used in the clinical setting for the differential diagnosis between adrenocortical carcinomas and non-functioning adenomas. Moreover, IGF2 was demonstrated to influence adrenocortical cancer cell proliferation, metabolism, and viability, but not cell invasiveness. Altogether, these data suggest that different IGF2 concentrations in ACC can be responsible for different biological behaviors.

## Supplementary information

Supplementary file 1
