# Supplementary material for: IGF2 role in adrenocortical carcinoma biology
Source: Endocrine. 2019 Aug 4;66(2):326–37. doi: 10.1007/s12020-019-02033-5 (PMC6838304; doi:10.1007/s12020-019-02033-5)
Supplement: Supplementary file 1 [file 12020_2019_2033_MOESM1_ESM.pdf]

<u>Supplementary File 1</u>

Manuscript title: IGF2 role in adrenocortical carcinoma biology

Journal: Endocrine

Authors: Sofia S. Pereira, Mariana P. Monteiro, Madalena M Costa, Ângela Moreira, Marco G. Alves, Pedro F. Oliveira, Ivana Jarak, Duarte Pignatelli

Corresponding author: Duarte Pignatelli (Instituto de Investigação e Inovação em Saúde (I3S), Universidade do Porto, Portugal; dpignatelli@ipatimup.pt)

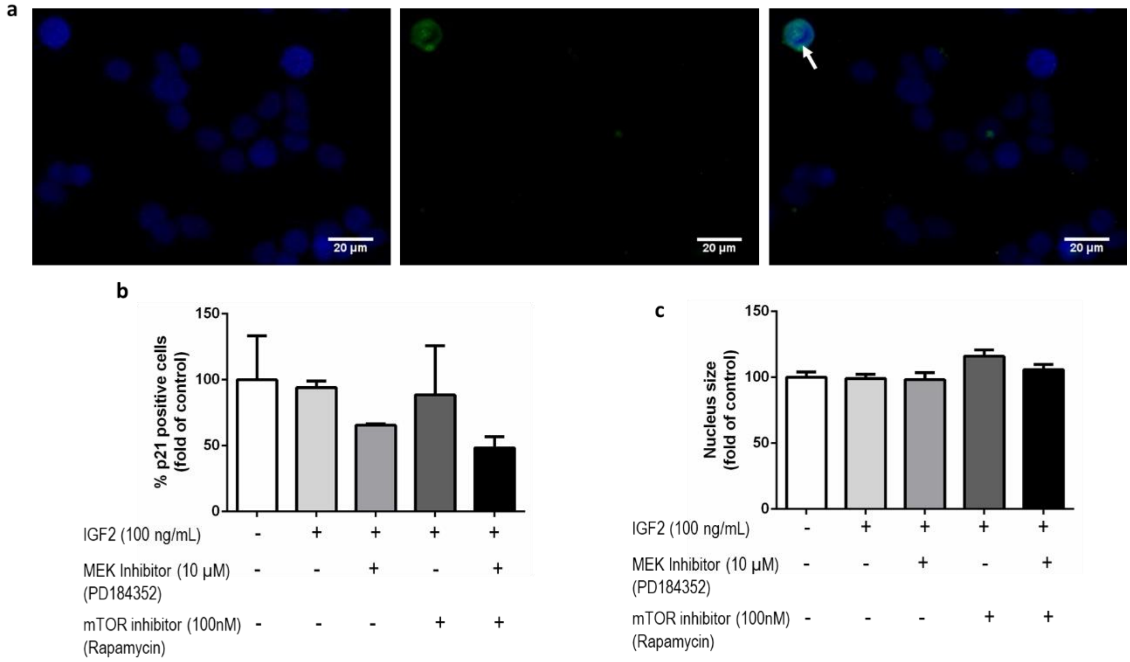

Supplementary File 2- H295R cells senescence assessed through p21 immunofluorescence (a and b) and nucleus size (c). a) Exemplificative images of p21 staining in H295R cells. The arrow indicates a p21 positive cell; b) percentage of p21 positive cells expressed in H295R cells after treatment with IGF2 (100ng/mL) with and without the pathway's inhibitors (100nM of Rapamycin for mTOR pathway inhibition and 10nM of PD184352 for MAPK pathway inhibition); b) nucleus size of H295R cells after treatment with IGF2 (100ng/mL) with and without the pathway's inhibitors (100nM of Rapamycin for mTOR pathway inhibition and 10nM of PD184352 for MAPK pathway inhibition).
